# Supplementary material for: Effect sizes of APOE e4 on the same general cognitive ability test taken by the same people from age 11 to age 90: The Lothian Birth Cohorts 1921 and 1936
Source: Mol Psychiatry. 2025 Oct 6;31(1):27–38. doi: 10.1038/s41380-025-03274-9 (PMC12700797; doi:10.1038/s41380-025-03274-9)
Supplement: Supplementary file 1 — Supplementary information for: Effect sizes of APOE e4 on the same general cognitive ability test taken by the same people from age 11 to age 90: The Lothian Birth Cohorts 1921 and 1936 [file 41380_2025_3274_MOESM1_ESM.docx]

**Supplementary information for:**

**Effect sizes of *APOE* e4 on the same general cognitive ability test taken by the same people from age 11 to age 90: The Lothian Birth Cohorts 1921 and 1936**

Ian J. Deary, Sarah E. Harris, Tom Russ, Simon R Cox, and Janie Corley

**Component loadings of the Moray House Test**

Oblique rotation of the first two principal components found a first component that accounted for 39.2% of the total variance and a second component accounting for 10.6% (Supplementary Figure 2); these two components had an absolute correlation of 0.39. Using the pattern matrix, the first had highest loadings for verbal knowledge and memory tests, with a Moray House Test loading of 0.67. On the second component, the four tests with loadings greater than 0.6 were assessments of processing speed, and the Moray House Test loaded 0.31.

**Table S1.** Correlations between Moray House Test (MHT) scores at each age with their standard errors

|  | **Lothian Birth Cohort 1936** | | | |
| --- | --- | --- | --- | --- |
| **Age** | **MHT 11** | **MHT 70** | **MHT 76** | **MHT 79** |
| MHT age 11 | 1 |  |  |  |
| MHT age 70 | .686 (0.024) | 1 |  |  |
| MHT age 76 | .637 (0.032) | .786 (0.025) | 1 |  |
| MHT age 79 | .635 (0.037) | .748 (0.031) | .830 (0.026) | 1 |
|  | **Lothian Birth Cohort 1921** | | | |
|  | **MHT 11** | **MHT 79** | **MHT 87** | **MHT 90** |
| MHT age 11 | 1 |  |  |  |
| MHT age 79 | .645 (0.035) | 1 |  |  |
| MHT age 87 | .506 (0.066) | .694 (0.051) | 1 |  |
| MHT age 90 | .527 (0.084) | .685 (0.067) | .845 (0.049) | 1 |

Note: Correlations are Pearson’s r values.

**Table S2**. Sensitivity analysis: main regression models of *APOE* e4 status on Moray House Test (MHT) score as presented in Table 3 but with exclusions for dementia

|  | **Lothian Birth Cohort 1936 (N = 903 at baseline)** | | | | | | | | | |
| --- | --- | --- | --- | --- | --- | --- | --- | --- | --- | --- |
|  |  | **Model 1 – age + sex** | | | |  | **Model 2 – age + sex + health covariates** | | | |
| **MHT** | **N** | **Std est** | **SE** | **P value** | **95% CI** | **N** | **Std est** | **SE** | **P value** | **95% CI** |
| Age 11 | 852 | 0.015 | 0.034 | 0.650 | -0.051, 0.081 |  | | | | |
| Age 70 | 898 | -0.012 | 0.033 | 0.714 | -0.077, 0.052 | 886 | -0.014 | 0.033 | 0.661 | -0.079, 0.050 |
| Age 76 | 559 | -0.020 | 0.042 | 0.633 | -0.102, 0.062 | 557 | -0.021 | 0.042 | 0.617 | -0.103, 0.061 |
| Age 79 | 419 | -0.098 | 0.047 | **0.039** | -0.191, 0.005 | 416 | -0.096* | 0.047 | **0.042** | -0.189, -0.004 |
|  | **Lothian Birth Cohort 1921 (N = 430 at baseline)** | | | | | | | | | |
| Age 11 | 382 | -0.005 | 0.050 | 0.915 | -0.103, 0.093 |  | | | | |
| Age 79 | 425 | -0.100 | 0.048 | **0.037** | -0.194, -0.006 | 419 | -0.097* | 0.047 | **0.042** | -0.189, -0.004 |
| Age 87 | 137 | -0.206 | 0.089 | **0.021** | -0.382, -0.031 | 135 | -0.189 | 0.090 | **0.036** | -0.365, -0.012 |
| Age 90 | 88 | -0.183 | 0.125 | 0.143 | -0.429, 0.062 | 88 | -0.220 | 0.121 | 0.069 | -0.457, 0.017 |

Note**:** The health covariates in model 2 are CVD, diabetes, stroke, and hypertension (yes/no). Dementia cases are those participants who have had dementia status ascertained by medical consensus any time between study baseline and the end of the follow-up period.

*The meta-analytic effect size across both cohorts at age 79 (adjusted model 2–see asterisked values above) is -0.097; SE = 0.033; P < 0.0037; 95%CI = -0.162, -0.031.

**Table S3**. Sensitivity analysis: main regression models of *APOE* e4 status on Moray House Test (MHT) score as presented in Table 4a for completers only but with exclusions for dementia

|  | **Lothian Birth Cohort 1936 (N = 409)** | | | | | | | | | |
| --- | --- | --- | --- | --- | --- | --- | --- | --- | --- | --- |
|  |  | **Model 1 – age + sex** | | | |  | **Model 2 – age + sex + health covariates** | | | |
| **MHT** | **N** | **Std est** | **SE** | **P value** | **95% CI** | **N** | **Std est** | **SE** | **P value** | **95% CI** |
| Age 11 | 383 | -0.042 | 0.051 | 0.409 | -0.141, 0.057 |  | | | | |
| Age 70 | 409 | -0.064 | 0.049 | 0.192 | -0.160, 0.032 | 409 | -0.062 | 0.049 | 0.209 | -0.158, 0.035 |
| Age 76 | 409 | -0.085 | 0.049 | 0.082 | -0.181, -0.011 | 409 | -0.083 | 0.049 | 0.088 | -0.179, 0.012 |
| Age 79 | 409 | -0.116 | 0.048 | **0.016** | -0.210, -0.021 | 406 | -0.115* | 0.048 | **0.017** | -0.210, -0.021 |
|  |  |  |  |  |  |  |  |  |  |  |
|  | **Lothian Birth Cohort 1921 (N = 87)** | | | | | | | | | |
| Age 11 | 76 | -0.087 | 0.108 | 0.422 | -0.298, 0.125 |  | | | | |
| Age 79 | 87 | -0.106 | 0.106 | 0.316 | -0.314, 0.101 | 87 | -0.114* | 0.106 | 0.283 | -0.321, 0.094 |
| Age 87 | 87 | -0.097 | 0.106 | 0.360 | -0.305, 0.111 | 87 | -0.121 | 0.110 | 0.269 | -0.336, 0.094 |
| Age 90 | 87 | -0.171 | 0.103 | 0.097 | -0.374, 0.031 | 87 | -0.185 | 0.102 | 0.071 | -0.385, 0.016 |

Note: Completers are those who attended, and sat the MHT, at all 3 waves in later life. The health covariates in model 2 are CVD, diabetes, stroke, and hypertension (yes/no). Dementia cases are those participants who have had dementia status ascertained by medical consensus any time between study baseline and the end of the follow-up period.

*The meta-analytic effect size across both cohorts at age 79 (adjusted model 2—see asterisked values above) is --0.115; SE = 0.044; P = 0.0086; 95%CI = -0.201, -0.029.

**Table S4**. Longitudinal growth models: associations of *APOE* e4 with change in Moray House Test (MHT) score across later life (fully-adjusted) with dementia exclusions

|  | **Lothian Birth Cohort 1936 (N = 903 at baseline)** | | | | | | | |
| --- | --- | --- | --- | --- | --- | --- | --- | --- |
|  | **Intercept (*APOE* e4 and MHT at baseline, age 70)** | | | | **Slope (*APOE* e4 and MHT change from age 70 to 79)** | | | |
| **Predictors** | **Std est** | **SE** | **P value** | **95% CI** | **Std est** | **SE** | **P value** | **95% CI** |
| *APOE* e4 | -0.014 | 0.035 | 0.693 | -0.083, 0.055 | -0.110 | 0.075 | 0.146 | -0.258, 0.038 |
| CFI | 1.000 |  | | | | | | |
| RMSEA | 0.000 |  |  |  |  |  |  |  |
| SRMR | 0.005 |  |  |  |  |  |  |  |
|  | **Lothian Birth Cohort 1921 (N = 430 at baseline)** | | | | | | | |
|  | **Intercept (*APOE* e4 and MHT at baseline, age 79)** | | | | **Slope (*APOE* e4 and MHT change from age 79 to 90)** | | | |
| *APOE* e4 | -0.097 | 0.048 | **0.045** | -0.191, -0.002 | -0.168 | 0.096 | 0.081 | -0.357, 0.021 |
| CFI | 0.988 |  | | | | | | |
| RMSEA | 0.026 |  |  |  |  |  |  |  |
| SRMR | 0.017 |  |  |  |  |  |  |  |

Note: The growth curve model for each cohort is fully-adjusted for all wave 1 covariates: age; sex; CVD; stroke; high blood pressure; diabetes. Slope values are based on change in Moray House Test score from age 70 to age 79 (for LBC1936) and from age 79 to age 90 (for LBC1921). Model fit was tested using absolute fit indices: Comparative Fit Index (CFI; values > 0.95 considered acceptable), Root Mean Square Error of Approximation (RMSEA; values < 0.06 considered acceptable), and Standardized Root Mean Square Residual (SRMR; values < 0.08 considered acceptable).

**Table S5**. Moray House Test (MHT) scores at each time-point according to *APOE* e4 genotype, for all comers and completers

|  | | **Lothian Birth Cohort 1936** | | | | | |
| --- | --- | --- | --- | --- | --- | --- | --- |
|  |  | **All comers** | | | ***Completers** | | |
|  |  | **N** | **M** | **SD** | **N** | **M** | **SD** |
| MHT at age 11 (childhood) | No e4 alleles | 671 | 49.3 | 11.2 | 300 | 51.9 | 10.4 |
|  | One e4 allele | 264 | 49.1 | 11.4 | 120 | 50.4 | 12.2 |
|  | Two e4 alleles | 19 | 51.2 | 12.1 | 10 | 54.9 | 10.0 |
|  | Total | 954 | 49.3 | 11.3 | 430 | 51.6 | 11.0 |
| MHT at age 70 (wave 1) | No e4 alleles | 705 | 64.7 | 8.0 | 320 | 67.0 | 6.4 |
|  | One e4 allele | 276 | 64.4 | 8.3 | 127 | 65.7 | 7.9 |
|  | Two e4 alleles | 20 | 63.2 | 7.3 | 10 | 66.9 | 4.9 |
|  | Total | 1001 | 64.6 | 8.0 | 457 | 66.7 | 6.8 |
| MHT at age 76 (wave 3) | No e4 alleles | 445 | 63.9 | 8.9 | 320 | 65.3 | 7.9 |
|  | One e4 allele | 178 | 61.1 | 10.7 | 127 | 62.7 | 9.9 |
|  | Two e4 alleles | 13 | 60.1 | 10.6 | 10 | 64.3 | 5.7 |
|  | Total | 636 | 63.0 | 9.6 | 457 | 64.5 | 8.6 |
| MHT at age 79 (wave 4) | No e4 alleles | 331 | 63.7 | 9.5 | 320 | 64.0 | 9.2 |
|  | One e4 allele | 129 | 60.3 | 10.5 | 127 | 60.6 | 10.3 |
|  | Two e4 alleles | 11 | 59.3 | 7.1 | 10 | 60.4 | 6.4 |
|  | Total | 471 | 62.7 | 9.8 | 457 | 63.0 | 9.6 |
|  | | **Lothian Birth Cohort 1921** | | | | | |
|  |  | **All comers** | | | ***Completers** | | |
|  |  | **N** | **M** | **SD** | **N** | **M** | **SD** |
| MHT at age 11 (childhood) | No e4 alleles | 354 | 46.90 | 12.1 | 82 | 50.11 | 10.9 |
|  | One e4 allele | 125 | 45.78 | 11.3 | 22 | 45.73 | 10.8 |
|  | Two e4 alleles | 4 | 47.25 | 7.0 | 0 | . | . |
|  | Total | 483 | 46.61 | 11.8 | 104 | 49.18 | 11.0 |
| MHT at age 79 (wave 1) | No e4 alleles | 394 | 60.17 | 10.2 | 94 | 63.91 | 8.3 |
|  | One e4 allele | 135 | 57.79 | 10.6 | 25 | 61.36 | 7.6 |
|  | Two e4 alleles | 4 | 47.75 | 12.9 | 0 | . | . |
|  | Total | 533 | 59.47 | 10.4 | 119 | 63.38 | 8.2 |
| MHT at age 87 (wave 3) | No e4 alleles | 153 | 55.79 | 12.8 | 94 | 59.07 | 10.6 |
|  | One e4 allele | 45 | 48.80 | 15.9 | 25 | 53.84 | 11.3 |
|  | Two e4 alleles | 0 | . | . | 0 | . | . |
|  | Total | 198 | 54.20 | 13.8 | 119 | 57.97 | 10.9 |
| MHT at age 90 (wave 4) | No e4 alleles | 95 | 53.72 | 14.1 | 94 | 54.04 | 13.8 |
|  | One e4 allele | 25 | 43.96 | 14.0 | 25 | 43.96 | 14.0 |
|  | Two e4 alleles | 0 | . | . | 0 | . | . |
|  | Total | 120 | 51.68 | 14.5 | 119 | 51.92 | 14.4 |

Note: *Completers are participants who attended all three waves of testing in older age (LBC1936: ages 70, 76, 79; LBC1921: ages 79, 87, 90) and completed the MHT during each of these waves.

For the LBC1936, exact ages in years (M±SD) were as follows: age 11 = 10.9±0.3; age 70 = 69.5±0.8; age 76 = 76.2±0.7; age 79 = 79.3±0.6.

For the LBC1921, exact ages in years (M±SD) were as follows: age 11 = 10.9±0.3; age 79 = 79.5±0.7; age 87 = 86.6±0.4; age 90 = 90.1±0.1.

**Table S6a**. Lothian Birth Cohort 1936: Comparisons between Moray House Test (MHT) scores according to *APOE* e4 genotype at each time-point, for all comers and completers

|  | **Lothian Birth Cohort 1936** | | | | | |
| --- | --- | --- | --- | --- | --- | --- |
|  | **All comers** | | | ***Completers** | | |
|  | **P** | **Eta squared** | **95% CI** | **P** | **Eta squared** | **95% CI** |
| MHT at age 11 (childhood) | 0.750 | 0.001 | 0.000, 0.006 | 0.264 | 0.006 | 0.000, 0.026 |
| MHT at age 70 (wave 1) | 0.646 | 0.001 | 0.000, 0.006 | 0.179 | 0.008 | 0.000, 0.028 |
| MHT at age 76 (wave 3) | **0.002** | 0.019 | 0.003, 0.043 | **0.018** | 0.018 | 0.000, 0.046 |
| MHT at age 79 (wave 4) | **0.002** | 0.026 | 0.004, 0.059 | **0.002** | 0.028 | 0.004, 0.061 |

Note: *Completers are participants who attended all three waves of testing in older age (LBC1936: ages 70, 76, 79) and completed the MHT during each of these waves.

For the LBC1936, exact ages in years (M±SD) were as follows: age 11 = 10.9±0.3; age 70 = 69.5±0.8; age 76 = 76.2±0.7; age 79 = 79.3±0.6.

Results are derived from a One-Way Analysis of Variance.

**Table S6b**. Lothian Birth Cohort 1936: Comparisons between Moray House Test (MHT) scores according to *APOE* e4 genotype at each time-point, for all comers and completers. Results are from Tukey HSD post-hoc tests following one-way ANOVA (see Table 5a)

| **Sample** | **Wave** | ***APOE* e4 genotype comparison** | **Mean Difference** | **Std. Error** | ***p*-value** | **95% CI** |
| --- | --- | --- | --- | --- | --- | --- |
| **All Comers** | 3 | No e4 vs 1 e4 allele | **2.779** | 0.842 | **.003** | 0.80 – 4.76 |
|  |  | No e4 vs 2 e4 alleles | 3.797 | 2.672 | .331 | -2.48 – 10.08 |
|  |  | 1 e4 vs 2 e4 alleles | 1.019 | 2.729 | .926 | -5.39 – 7.43 |
|  | 4 | No e4 vs 1 e4 allele | **3.389** | 1.008 | **.002** | 1.02 – 5.76 |
|  |  | No e4 vs 2 e4 alleles | 4.434 | 2.978 | .297 | -2.57 – 11.44 |
|  |  | 1 e4 vs 2 e4 alleles | 1.045 | 3.052 | .937 | -6.13 – 8.22 |
| **Completers*** | 3 | No e4 vs 1 e4 allele | **2.544** | 0.891 | **.012** | 0.45 – 4.64 |
|  |  | No e4 vs 2 e4 alleles | 0.953 | 2.729 | .935 | -5.46 – 7.37 |
|  |  | 1 e4 vs 2 e4 alleles | -1.591 | 2.791 | .836 | -8.15 – 4.97 |
|  | 4 | No e4 vs 1 e4 allele | **3.461** | 0.996 | **.002** | 1.12 – 5.80 |
|  |  | No e4 vs 2 e4 alleles | 3.644 | 3.049 | .457 | -3.53 – 10.81 |
|  |  | 1 e4 vs 2 e4 alleles | 0.183 | 3.119 | .998 | -7.52 – 7.15 |

Note: *Completers are participants who attended all three waves of testing in older age

**Table S7**. Lothian Birth Cohort 1936: *APOE* e4 status associations with dropouts, deaths, and dementia

|  | **Lothian Birth Cohort 1936** | | | | |
| --- | --- | --- | --- | --- | --- |
|  | ***APOE*e4, yes** | ***APOE*e4, no** | **Total** | **Pearson**  **Chi-square** | **P value** |
| **Dropout status** |  |  |  |  |  |
| Dropouts | 148 | 351 | 499 | 0.001 | 0.974 |
| Non-dropouts | 152 | 359 | 511 |  |  |
| Total | 300 | 710 | 1010 |  |  |
| **Death status** |  |  |  |  |  |
| Dead | 74 | 169 | 243 | 0.089 | 0.766 |
| Alive | 225 | 539 | 764 |  |  |
| Total | 299 | 708 | ^1^1007 |  |  |
| **Dementia status** |  |  |  |  |  |
| Dementia | 62 | 45 | 107 | 46.145 | **<0.001** |
| No dementia | 179 | 519 | 698 |  |  |
| Total | 241 | 564 | ^2^805 |  |  |
|  | **Lothian Birth Cohort 1921** | | | | |
|  | ***APOE*e4, yes** | ***APOE*e4, no** | **Total** | **Pearson**  **Chi-square** | **P value** |
| **Dropout status** |  |  |  |  |  |
| Dropouts | 115 | 298 | 413 | 1.566 | 0.211 |
| Non-dropouts | 28 | 98 | 126 |  |  |
| Total | 413 | 126 | 539 |  |  |
| **Death status** |  |  |  |  |  |
| Dead | 101 | 233 | 334 | 5.743 | **0.017** |
| Alive | 42 | 160 | 202 |  |  |
| Total | 143 | 393 | ^3^536 |  |  |
| **Dementia status** |  |  |  |  |  |
| Dementia | 43 | 66 | 109 | 11.699 | **<0.001** |
| No dementia | 100 | 330 | 430 |  |  |
| Total | 143 | 396 | 539 |  |  |

Note: Dropouts (yes/no) were defined as those who took part at the baseline assessment at wave 1 but did not return to the study at any point before the end of wave 4 (the last measurement period in this study) regardless of whether they sat the MHT at all three waves in later life (i.e., ‘completers’).

Deaths (yes/no) were defined as those for whom death certificates had been obtained via mortality linkage data with National Records Scotland by the end of the last testing wave included in this study (1936, wave 4, ~end 2012; 1921, wave 4, ~ end 2017). ^1^N=3 LBC1936 participants and ^3^N=3 LBC1921 participants were unable to be traced for mortality linkage (due to moving outwith Scotland) and were coded as missing data. ^2^N=205 LBC1936 participants were unable to be screened for dementia due to attending wave 1 only (specific consent to link to medical records was taken only from wave 2 onwards) and coded as missing data.

Dementia status (yes/no) was ascertained by medical consensus between study baseline and the end of the follow-up period.

Results are derived from Chi-square tests.


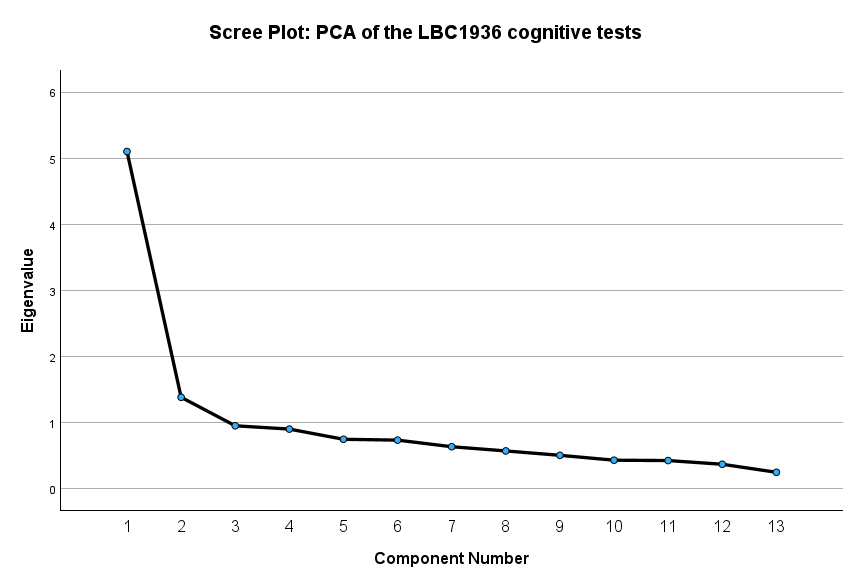


**Figure S1.** Scree plot for the principal component analysis (PCA) of all 14 individual cognitive tests administered at wave 1 (baseline) in the LBC1936 study. National Adult Reading Test (NART) and Wechsler Test of Adult Reading (WTAR) tests were averaged to produce a single score.

**
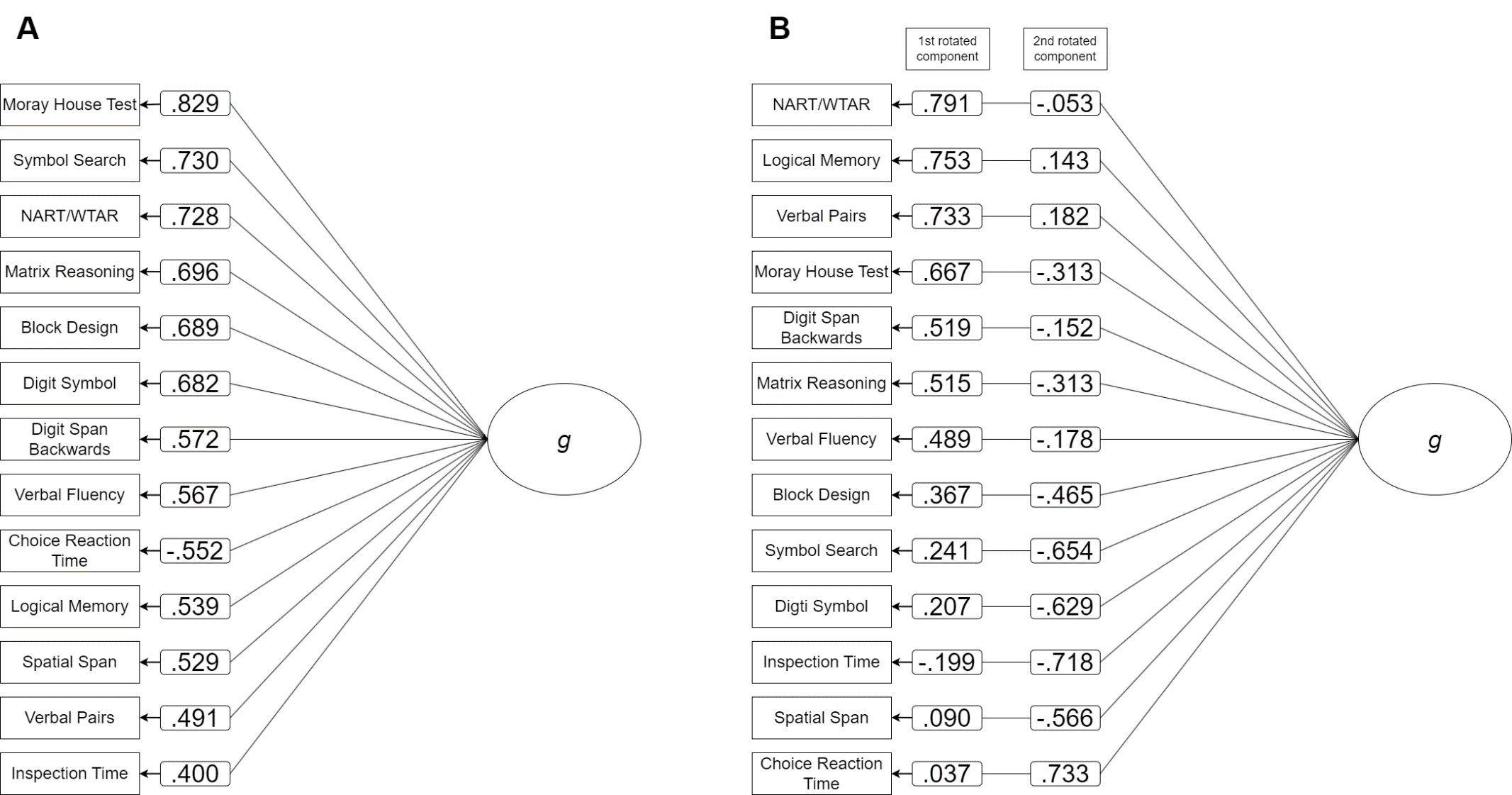
**

**Figure S2**. Principal components analysis (PCA) of all 14 cognitive tests in the Lothian Birth Cohort 1936. National Adult Reading Test (NART) and Wechsler Test of Adult Reading (WTAR) tests were averaged to produce a single score. **A** presents the 1st unrotated component and the loadings of each cognitive test on this component (*g*) from highest to lowest. The total variance accounted for by *g* by the first unrotated component is 39%. **B** presents the rotated components (using oblique rotation) and the factor loadings of each cognitive test from highest to lowest for the 1st rotated component. Total variance accounted for by the two rotated components is 50% (39.2% for the 1st component and 10.6% for the 2nd component).
